# Quality of diabetes care in cancer: a systematic review

**DOI:** 10.1093/intqhc/mzy124

**Published:** 2018-06-15

**Authors:** Robert I Griffiths, Nancy L Keating, Clare R Bankhead

**Affiliations:** 1 Nuffield Department of Primary Care Health Sciences, University of Oxford, Oxford, UK; 2 Division of General Internal Medicine, Department of Medicine, Johns Hopkins University School of Medicine, Baltimore, MD, USA; 3 Department of Health Care Policy, Harvard Medical School, Boston, MA, USA; 4 Division of General Internal Medicine and Primary Care Research, Department of Medicine Research, Brigham and Women’s Hospital, Boston, MA, USA

**Keywords:** systematic review, diabetes mellitus, neoplasms, quality of care

## Abstract

**Purpose:**

Overlooking other conditions during cancer could undermine gains associated with early detection and improved cancer treatment. We conducted a systematic review on the quality of diabetes care in cancer.

**Data sources:**

Systematic searches of Medline and Embase, from 1996 to present, were conducted to identify studies on the quality of diabetes care in patients diagnosed with cancer.

**Study selection:**

Studies were selected if they met the following criteria: longitudinal or cross-sectional observational study; population consisted of diabetes patients; exposure consisted of cancer of any type and outcomes consisted of diabetes quality of care indicators, including healthcare visits, monitoring and testing, control of biologic parameters, or use of diabetes and other related medications.

**Data extraction:**

Structured data collection forms were developed to extract information on the study design and four types of quality indicators: physician visits, exams or diabetes education (collectively ‘healthcare visits’); monitoring and testing; control of biologic parameters; and medication use and adherence.

**Results of data synthesis:**

There were 15 studies from five countries. There was no consistent evidence that cancer was associated with fewer healthcare visits, lower monitoring and testing of biologic parameters or poorer control of biologic parameters, including glucose. However, the weight of the evidence suggests cancer was associated with lower adherence to diabetes medications and other medications, such as anti-hypertensives and cholesterol-lowering agents.

**Conclusion:**

Evidence indicates cancer is associated with poorer adherence to diabetes and other medications. Further primary research could clarify cancer’s impact on other diabetes quality indicators.

## Introduction

Early detection and advances in therapy and supportive care have substantially improved the relative survival of many of the most common types of cancer [1]. Consequently, overall morbidity and mortality in cancer seem to depend increasingly on the quality and outcomes of care for underlying conditions [2]. In response, leading cancer organizations in the UK, including Cancer Research UK and Macmillan Cancer Support, have expressed concern that overlooking other medical conditions during cancer treatment and follow-up could result in excess morbidity and mortality, thereby undermining gains associated with early detection and improved treatment of cancer [3, 4].

Although cancer could have an adverse impact on many conditions, and vice versa, the quality of diabetes care for patients with cancer deserves particular attention for the following reasons. First, diabetes and cancer are common, especially in older people. Second, diabetes and some types of cancer, including breast and colorectal, co-occur at rates that are higher than expected by chance alone, which implies shared risk factors and, possibly, causal mechanisms [5]. Third, some types of cancer treatments, for instance, androgen deprivation therapy for prostate cancer [6], appear to increase the risk of diabetes and related complications, and may worsen diabetes control. Fourth, diabetes is associated with excess morbidity and mortality in cancer [7].

We conducted a systematic review to address the following ‘patients, exposures, outcomes’ (PEO) research question: among patients with diabetes, does a diagnosis of cancer impact the quality of diabetes care?

## Methods

The Methods and Results sections are reported according to the 2009 Preferred Reporting Items for Systematic Reviews and Meta-Analyses (PRISMA) guidelines [8, 9].

### Protocol

A protocol (available upon request) was developed by the co-authors, with input from external reviewers (N.F.K. and C.F.S.: see Acknowledgements), each of whom had a paper included in this review.

### Eligibility criteria

The ‘patients’ of interest were those diagnosed with type I or type II diabetes, including (A) those with diabetes that preceded cancer, and (B) those with diabetes that may have been diagnosed after cancer, but before the beginning of the observation period for outcomes. The ‘exposure’ of interest was cancer of any type; and the ‘outcomes’ of interest were diabetes quality of care measures. Several types of observational cohort studies were considered for inclusion: longitudinal cohort studies in which patients with pre-existing diabetes were followed from before to after diagnosis of cancer for changes in diabetes quality of care; longitudinal cohort studies in which patients with pre-existing diabetes were followed from the time of cancer diagnosis; longitudinal studies in which patients with diabetes were followed from a specific time point, e.g. 3 years, after diagnosis of cancer; and cross-sectional studies in which patients with underlying diabetes and cancer were observed during a discrete time interval, e.g. one calendar year, after the diagnoses of diabetes and cancer had been established.

Studies were eligible for inclusion if they reported on diabetes quality of care indicators, including physician visits and examinations, monitoring and testing of biologic parameters including glucose, blood pressure and lipids, control of those biologic parameters, or medication use (diabetes and others including anti-hypertensives and lipid-lowering agents, which are important in preventing diabetes complications).

### Information sources

Using an OVID platform, systematic searches of Medline and Embase, from 1996 to the present (9/2016), were conducted to identify studies on the quality of diabetes care in patients diagnosed with cancer. Access to translation services was not available. Therefore, only English language articles, and only those published between 1996 and the present, as it has been argued that this period constitutes the era of modern diabetes care [10], were considered for inclusion in the review. The searches were conducted only in Medline and Embase based on findings from a previous study that showed these two databases are sufficient for identifying English language papers on diabetes epidemiology [11]. In addition, the bibliographies of those articles retrieved for review were searched, and three authors were contacted to request relevant references they might have in their research bibliographies.

### Search

The search began by tabulating Medical Subject Headings (MeSH) terms [12] from eight articles [13–20] identified while preparing the protocol for the systematic review, which were considered to address the PEO statement for this review. Second, those MeSH terms shared in common across these eight articles were identified. The patients of interest were those diagnosed with diabetes. However, not all of these articles included ‘diabetes’ or some derivative of ‘diabetes’ as a MeSH term. Therefore, MeSH terms were added for ‘chronic disease’ and ‘comorbidity’ to the search terms for patients. The exposure of interest was cancer. All eight articles included a MeSH term either for ‘neoplasms’ or for ‘carcinoma.’ Therefore, these were used to identify the exposure of interest. All eight articles included a MeSH term for ‘quality,’ ‘disease management’ or ‘disease progression.’ These were used to identify studies reporting outcomes of potential interest for the review. MeSH terms for survival or for other clinical outcomes of diabetes or cancer were not included, as these outcomes were beyond the scope of the review.

A preliminary search based on the strategy above produced in excess of 20 000 articles. Therefore, in order to narrow the search, the next step was to examine the titles of the original eight articles and identify keywords shared in common. As a result, the search (Supplemental Materials Box A) was subsequently restricted to those articles with both ‘cancer’ and any of ‘cormorbid’ or ‘diabetes’ or ‘chronic’ in the title.

### Study selection

Screening studies for selection consisted of reviewing titles and abstracts of all articles obtained through the Medline and Embase searches described above. The following inclusion criteria were used: (1) longitudinal or cross-sectional observational study; (2) population consisted of diabetes patients; (3) exposure consisted of cancer of any type and (4) outcomes consisted of quality of diabetes care indicators, including healthcare visits, monitoring and testing of biologic parameters, control of biologic parameters or use of diabetes and other medications considered important for preventing diabetes complications, including anti-hypertensives and lipid-lowering agents. No additional articles were identified through contacts with the authors.

### Data collection process and data items

A structured data collection form was developed to abstract information on the design of each study included in the systematic review, including the overall study design, country of origin, data source(s), patients, study enrolment period, length of follow-up, outcome measures and methods of adjustment. Also, a structured form was developed for each category of outcomes, consisting of (1) physician visits, exams or diabetes education (collectively ‘healthcare visits’), (2) monitoring and testing of biologic parameters, (3) control of biologic parameters and (4) medication use and adherence. Using these forms, each outcome result was classified as one of the following: (A) better in cancer than controls; or (B) no different between cancer patients and controls; or (C) worse in cancer patients than controls. Assignment was based on the statistical significance of observed differences in quality indicators, either after compared to before diagnosis in single cohorts of cancer patients, or between cancer patients and controls. Outcomes were extracted by two reviewers (R.I.G. and R.J.H.), with concordance of 97% (83/86 outcomes).

### Quality assessment

A quality score was assigned to each article using the Newcastle–Ottawa Quality Assessment Scale for Cohort Studies [21]. The maximum score for a longitudinal cohort study that included a control group, with either matching or statistical adjustment for potential confounding, was eight stars; the maximum score for a longitudinal study with a ‘before and after’ design that included only an exposure group, i.e. patients with cancer, was five stars; and the maximum score for a cross-sectional study with a control group was seven stars.

### Risk of bias in individual studies

The risk of bias in individual studies was examined using questions from an item bank developed for the US Agency for Healthcare Research and Quality to assess the risk of bias and confounding for observational studies of interventions or exposures [22] (Supplemental Materials, Box B).

### Summary measures

For binary outcomes, summary measures included proportions/percentages with 95% confidence intervals (CIs) as well as risk ratios (RR)/odds ratios (OR) with 95% CIs. For binary time-to-event variables, hazard ratios (HR) with 95% CIs were included wherever possible. For continuous variables, summary measures consisted of means and 95% CIs. In instances where they were not reported, attempts were made to calculate the summary measures using other information in the study, e.g. cross-tabulations of exposure (cancer/control) by outcome [23]. When this approach failed (four studies), the lead author of the study was contacted for the information.

### Synthesis of results

Performing a formal synthesis of the findings was considered. However, there was considerable heterogeneity in the design of the studies included in the systematic review, including the types of cohort studies, types of cancers, length of follow-up and specification of the outcomes variables. Also, several studies did not contain sufficient information to pool findings. Usually, this was due to the fact that diabetes patients comprised only a subset of the patient population, and precise information on the size of the subset was not available directly through contacting the lead authors of those studies. Therefore, a formal synthesis of the results was not undertaken.

## Results

### Study selection

**Figure 1:**
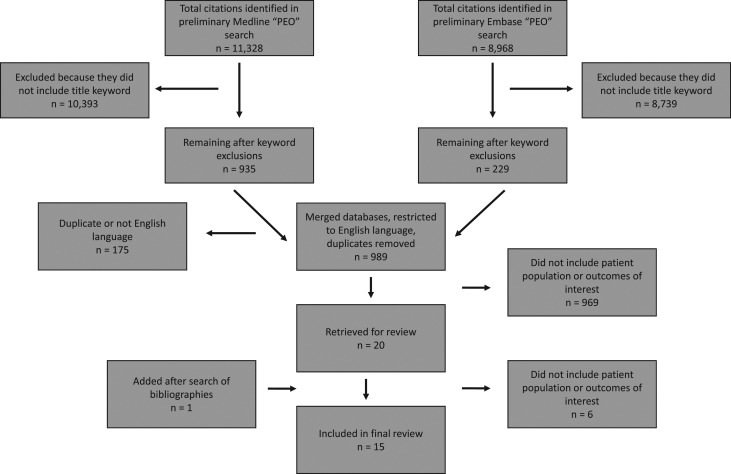
Flowchart of search results.

The preliminary searches in Medline and Embase, which were based on MeSH terms alone, resulted in identifying 11 328 and 8 968 articles, respectively. After applying the title keyword exclusion criteria, eliminating duplicates and excluding non-English language articles, 989 remained for preliminary review, of which 20 were retrieved for full review, and one additional article was identified from a search of the bibliographies. Upon full review, six were found not to include either the patient population or an outcome of interest, leaving 15 articles [13–20, 24–30] for inclusion (Fig. 1).

### Study characteristics

There were six (40%; 95% CI, 34–46%) longitudinal cohort studies in which patients with pre-existing diabetes were followed from before to after incident diagnosis of cancer [15, 16, 24–27], six (40%; 95% CI, 34–46%) longitudinal cohort studies in which patients with diabetes were followed only after cancer [13, 14, 17, 18, 20, 28] and three (20%; 95% CI, 15–25%) retrospective cross-sectional studies in which patients with historical diagnoses of diabetes and cancer were assessed for quality of care during a fixed window of time (usually one calendar year) after both diagnoses [19, 29, 30] (Supplemental Materials, Table A). The majority of studies (10/15: 67%; 95% CI, 61–73%) were from the USA [13–16, 18–20, 24, 25, 27], with two from the Netherlands [26, 28], and one each from Australia [30], Korea [29] and the UK [17]. The most common types of cancers studied were breast (8/15: 53%; 95% CI, 47–60%) [13, 15, 17, 18, 24, 25, 27, 30], colorectal (5/15: 33%; 95% CI, 27–39%) [13, 15–17, 20] and prostate (4/15: 27%; 95% CI, 21–32%) [13, 15, 17, 30].

### Quality and risk of bias

Overall, study quality was high (Supplemental Materials, Table B and C). The main reason studies failed to achieve the maximum allowed score was that they were not necessarily representative of the national population of cancer patients, according to types of cancers included, patient age and/or geographic representation. The results of the analysis of bias within individual studies (Supplemental Materials, Table D) were consistent with the quality analyses.

### Diabetes quality of care indicators

#### Healthcare visits

Seven studies reported on healthcare visits (Table 1): six from the USA [13, 14, 16, 18–20] and one from the Netherlands [28]. Outcome measures consisted primarily of the rates/proportions of general practitioner/other visits [13, 16, 20] and eye exams to assess for retinopathy [13, 16, 18–20], with one study reporting on diabetes education [14]. Overall, observed differences between cancer patients and controls were small and/or not statistically significant (Table 1, centre column). Plots of RRs (Supplemental Materials, Figure A), and rates of visits/exams before compared to after cancer diagnosis (Supplemental Materials, Figure B), further illustrate these findings.

**Table 1:** Healthcare visits

Study	Difference between cancer patients and controls		
	Better in cancer than controls	No difference	Worse in cancer than controls
	(or ‘after’ compared to ‘before’ cancer depending on the study design)	(or ‘after’ compared to ‘before’ cancer depending on the study design)	(or ‘after’ compared to ‘before’ cancer depending on the study design)
Heins (2015) [28]	Cancer patients had more general practitioner CONTACTS FOR ANY REASON per year than controls (cancer, mean 11.9; 95% confidence interval (CI) 10.9–12.9 versus control, mean 10.0; 95% CI, 9.5–10.5). Cancer Patients had more general practitioner CONTACTS FOR CONDITIONS OTHER THAN DIABETES per year than controls (cancer, mean 8.3; 95% CI, 7.6–9.0 versus control, mean 7.1; 95% CI, 6.7–7.5).	There was no difference in the number of general practitioner CONTACTS FOR DIABETES per year between cancer patients and controls (cancer, mean 2.7; 95% CI, 2.4–3.0 versus control, mean 2.9; 95% CI, 2.7–3.1).	
		
Snyder (2013) [13]	Overall, a higher percent of all cancer (breast cancer [BC], colorectal cancer [CC], and prostate cancer [PC] combined) patients than controls had a 6-MONTHLY VISIT (cancer, 86%; 95% CI, 84–88% versus control, 81%; 95% CI, 80–82%). A higher percent of BC patients than controls had a 6-MONTHLY VISIT (cancer, 92%; 95% CI, 89–95% versus control, 84%; 95% CI, 81–87%). A higher percent of prostate cancer PC patients than controls had a 6-MONTHLY VISIT (cancer, 84%; 95% CI, 82–86% versus control, 76%; 95% CI, 74%–78%).	There was no difference between the percent of CC patients and controls who had a 6-MONTHLY VISIT (cancer, 83%; 95% CI, 80–86% versus control, 86%; 95% CI, 84–88%). Overall, there was no difference between the percent of all cancer patients and controls who had an ANNUAL EYE EXAM (cancer, 45%; 95% CI, 43–47% versus control, 46%; 95% CI, 44–48%). There was no difference between the percent of BC patients and controls who had an ANNUAL EYE EXAM (cancer, 49%; 95% CI, 44–54% versus control, 52%; 95% CI, 48–56%).	A lower percent of CC patients than controls had an ANNUAL EYE EXAM (cancer, 42%; 95% CI, 38–46% versus control, 49%; 95% CI, 46–52%).
		There was no difference between the percent of PC patients and controls who had an ANNUAL EYE EXAM (cancer, 45%; 95% CI, 42–48% versus control, 43%; 95% CI, 41–45%).	
Irizarry (2013) [14]		There was no difference between the percent of cancer patients and controls receiving DIABETES EDUCATION (cancer, 3.5% versus control, 3.8% [insufficient data to calculate Cis]).	
Chiao (2010) [16]		There was no difference between the rate (per patient) of PRIMARY CARE VISITS per year before and after CC diagnosis (mean before, 4.1; 95% CI, 3.6–4.6 versus mean after, 4.6; 95% CI, 4.0–5.2).	
		There was no difference between the rate (per patient) of EYE CLINIC VISITS per year before and after CC diagnosis (mean before, 1.2; 95% CI, 0.9–1.6 versus mean after, 1.2; 95% CI, 0.9–1.6).	
Hanchate (2010) [18]		There was no difference between the percent of cancer patients and controls receiving a BIENNIAL EYE EXAM (cancer, 58% versus control, 57% [insufficient data to calculate Cis]).	
Keating (2007) [19]		There was no difference between the percent of cancer patients and controls who had a DILATED RETINAL EXAM (cancer, 68% versus control, 67% [insufficient data to calculate Cis]).	
Earle (2004) [20]			A lower percent of CC patients than controls had a 6-MONTHLY VISIT (cancer, 93%; versus control 95% [insufficient data to calculate Cis]).
			A lower percent of CC patients than controls had an ANNUAL EYE EXAM (cancer 27% versus control, 30% [insufficient data to calculate Cis]).

Shaded results indicate that the findings were aggregated across multiple cancers, by the authors of that study, in instances where results from individual cancers included in that study also were reported.

#### Monitoring and testing of biologic parameters

Six studies reported on patterns of monitoring and testing of biologic parameters (Table 2): five from the USA [13, 16, 18–20] and one from the UK [17]. Monitoring and testing consisted of blood pressure checks [16, 17], cholesterol testing [16–19] and HbA1c/fructosamine testing [13, 16–20]. Several studies reported a mixture of non-statistically significant differences between cancer patients and controls and statistically significantly lower rates of monitoring and testing in cancer patients than controls.

**Table 2:** Monitoring and testing of biologic parameters

Study	Difference between cancer patients and controls		
	Better in cancer than controls	No difference	Worse in cancer than controls
	(or ‘after’ compared to ‘before’ cancer depending on the study design)	(or ‘after’ compared to ‘before’ cancer depending on the study design)	(or ‘after’ compared to ‘before’ cancer depending on the study design)
Snyder (2013) [13]		Overall, there was no difference between the percent of all cancer patients (breast cancer [BC], colorectal cancer [CC] and prostate cancer [PC] combined) and controls who had a 6-MONTHLY TEST FOR GLYCOSYLATED HAEMOGLOBIN (HbA1c) OR FRUCTOSAMINE (cancer, 27%; 95% confidence interval [CI], 25–29% versus control, 28%; 95% CI, 27–29%).	A lower percent of CC patients than controls had a 6-MONTHLY TEST FOR HbA1c OR FRUCTOSAMINE (cancer, 26%; 95% CI, 23–29% versus control, 31%; 95% CI, 28–34%).^a^
		There was no difference between the percent of BC patients and controls who had a 6-MONTHLY TEST FOR HbA1c OR FRUCTOSAMINE (cancer, 26%; 95% CI, 22–30% versus control, 29%; 95% CI, 26–32%).	
		There was no difference between the percent of PC patients and controls who had a 6-MONTHLY TEST FOR HbA1c OR FRUCTOSAMINE (cancer, 28%; 95% CI, 25–31% versus control, 27%; 95% CI, 25–29%).	
Chiao (2010) [16]		There was no difference in the rates (per patient-per year) of BLOOD PRESSURE CHECKS before compared to after CC diagnosis (mean before, 3.9; 95% CI, 3.4–4.4 versus mean after, 3.9; 95% CI, 3.4–4.5).	The rate of LOW DENSITY LIPOPROTEIN (LDL) CHECKS (per patient-per year) was higher before compared to after CC diagnosis (mean before, 1.4; 95% CI, 1.2–1.6 versus mean after, 1.1; 95% CI, 0.9–1.2).
		There was no difference in the rates (per patient-per year) of HbA1c CHECKS before compared to after CC diagnosis (mean before, 2.0; 95% CI, 1.8–2.2 versus mean after, 1.9; 95% CI, 1.6–2.1).	
Khan (2010) [17]		There was no difference between the percent of CC patients and controls who had a BLOOD PRESSURE MONITORING TEST OVER 3 YEARS (cancer, 90%; 95% CI, 84–94% versus control, 92%; 95% CI, 87–96%).	A lower percent of BC patients than controls received a BLOOD PRESSURE MONITORING TEST OVER 3 YEARS (cancer, 91%; 95% CI, 87–94% versus control, 95%; 95% CI, 92–97%).^a^
		There was no difference between the percent of BC patients and controls who had an HbA1c TEST OVER 3 YEARS (cancer, 86%; 95% CI, 82–90% versus control, 89%; 85–92%). There was no difference between the percent of CC patients and controls who had an HbA1c TEST OVER 3 YEARS (cancer, 83%; 95% CI, 77–89% versus control, 87%; 81–92%).	A lower percent of PC patients than controls received a BLOOD PRESSURE MONITORING TEST OVER 3 YEARS (cancer, 87%; 95% CI, 81–91% versus control, 94%; 95% CI, 90–97%).^a^ A lower percent of BC patients than controls received a CHOLESTEROL MONITORING TEST OVER 3 YEARS (cancer, 84%; 95% CI, 80–88% versus control, 91%; 95% CI, 87–94%).^a^
			A lower percent of CC patients than controls received a CHOLESTEROL MONITORING TEST OVER 3 YEARS (cancer, 80%; 95% CI, 73–85% versus control, 90%; 95% CI, 84–94%).^a^
			A lower percent of PC patients than controls received a CHOLESTEROL MONITORING TEST OVER 3 YEARS (cancer, 80%; 95% CI, 73–85% versus control, 90%; 95% CI, 84–94%).^a^
			A lower percent of PC patients than controls received an HbA1c MONITORING TEST OVER 3 YEARS (cancer, 80%; 95% CI, 74–86% versus control, 90%; 85–94%).^a^
Hanchate (2010) [18]		There was no difference between the percent of BC patients and controls who received a BIENNIAL LIPID TEST (cancer, 61% versus control, 61% [insufficient data to calculate CIs]).	
		There was no difference between the percent of BC patients and controls who received an ANNUAL HbA1c TEST (cancer, 34% versus control, 36% [insufficient data to calculate CIs]).	
Keating (2007) [19]	A higher percent of cancer patients than controls had an HbA1c TEST IN THE PAST 6 MONTHS (cancer, 66% versus control, 64% [insufficient data to calculate CIs]).	There was no difference between the percent of cancer patients and controls who received a LDL CHOLESTEROL TEST IN THE PAST YEAR (cancer, 85% versus control, 84% [insufficient data to calculate CIs]).	
	A higher percent of cancer patients than controls had a MICROALBUMIN TEST IN THE PAST YEAR (cancer, 59% versus control, 55% [insufficient data to calculate CIs]).		
Earle (2004) [20]		There was no difference between the percent of cancer patients and controls with a 6-MONTHLY HbA1c OR FRUCTOSAMINE TEST (cancer, 24 versus control, 26% [insufficient data to calculate CIs]).	

^a^Authors reported these differences as statistically significant at *P* < 0.05. In the table, the calculated 95% confidence intervals for the proportions overlap. However, the corresponding risk ratios calculated and reported in Figure 2.4 were all statistically significantly <1.0. Therefore, it is likely that the authors assessed the statistical significance of differences between cancer patients and controls using the risk ratio approach.

Shaded results indicate that the findings were aggregated across multiple cancers, by the authors of that study, in instances where results from individual cancers included in that study also were reported.

There was a relatively narrow distribution of RRs around 1.0, indicating that the proportions of cancer patients and controls receiving testing were quite similar (Supplemental Materials, Figure C). Plots of the per patient-per year rates of cancer patients with monitoring/testing, before compared to after cancer diagnosis, illustrate that, for the most part, changes were small (Supplemental Materials, Figure B).

#### Control of biologic parameters

Seven studies [15–17, 19, 27, 29, 30] reported on the control of blood pressure [15–17, 19], cholesterol [15–17, 19] and HbA1c [15–17, 19, 27, 29, 30] (Table 3). As with other measures reported above, evidence that cancer had an adverse impact on blood pressure, cholesterol and HbA1c control was inconsistent across cancer types and measures, both within and across the seven studies. Again, there was a relatively narrow distribution of RRs around 1.0, indicating that the proportions of cancer patients and controls with adequate control were quite similar (Supplemental Materials, Figure D).

**Table 3:** Control of biologic parameters

	Difference between cancer patients and controls		
Study	Better in cancer than controls	No difference	Worse in cancer than controls
	(or ‘after’ compared to ‘before’ cancer depending on the study design)	(or ‘after’ compared to ‘before’ cancer depending on the study design)	(or ‘after’ compared to ‘before’ cancer depending on the study design)
Calip (2015) [27]		Among patients with a medication possession ratio (MPR) <80, relative to the year before breast cancer (BC) diagnosis (glycosylated haemoglobin [HbA1c], 7.32: 95% Confidence Interval [CI], 7.01–7.63), MEAN HbA1c was similar in each of four periods after cancer diagnosis: treatment period (HbA1c = 7.46: 95% CI, 7.30–7.60); year +1 (HbA1c = 7.52: 95% CI, 7.36–7.68); year +2 (HbA1c = 7.53:95% CI, 7.37–7.69) and year +3 (HbA1c=7.42: 95% CI, 7.28–7.56).	Among all patients (those with MPR ≥80 plus those with MPR < 80), relative to the year before BC diagnosis (HbA1c [%], 6.96: 95% CI, 6.80–7.12), MEAN HbA1c was higher in each of four periods after cancer diagnosis: treatment period (HbA1c = 7.32: 95% CI, 7.18–7.46); year +1 (HbA1c = 7.41: 95% CI, 7.27–7.55); year +2 (HbA1c = 7.42: 95% CI, 7.28–7.56) and year +3 (HbA1c = 7.30: 95% CI, 7.17–7.43). Among patients with a MPR ≥80, relative to the year before BC diagnosis (HbA1c = 6.45: 95% CI, 6.35–6.55), MEAN HbA1c was higher in each of four periods after cancer diagnosis: treatment period (HbA1c = 6.83: 95% CI, 6.67–6.99); year +1 (HbA1c = 6.90: 95% CI, 6.75–7.05); year +2 (HbA1c = 6.96: 95% CI, 6.79–7.13) and year +3 (HbA1c = 6.96: 95% CI, 6.80–7.12).
Shin (2014) [29]		There was no difference between the percent of cancer patients and controls achieving ADEQUATE GLYCAEMIC CONTROL (HbA1c <7%): cancer survivors (25.2%: 95% CI, 17.5–34.8%); non-cancer, chronic disease controls (29.5%: 95% CI, 25.6–33.6%) and non-cancer, non-chronic disease controls (18.7%: 95% CI, 15.1–22.8%).	
Onitilo (2013) [30]		There was no difference in the MEDIAN HbA1c result score between patients with a history of BC and those without BC (cancer, score = 2 [HbA1c range 6.5–7.0%] interquartile range [IQR] = 1–3 versus control, score = 2; IQR = 1–3).	
		There was no difference in the MEDIAN HbA1c result score between patients with a history of PC and those without PC (cancer, score = 2; IQR = 1–3 versus control, score = 2; IQR = 1–3).	
Bayliss (2011) [15]	MEAN LOW DENSITY LIPOPROTEIN (LDL) CHOLESTEROL (mmol/l) decreased over 6 time periods from before to after cancer diagnosis (−24 to −6 months, mean 101; 95% CI, 98–104: −6 to 0 months, mean 98; 95% CI, 95–101: 0 to 6 months, mean 96; 95% CI, 92–100: 6–12 months, mean, 95; 95% CI, 91–99%: 12–24 months, mean 92; 95% CI, 89–95%: 24–60 months, mean 85, 95% CI, 82–89%).	There were no changes in MEAN HBA1C (%) over 6 time periods from before to after cancer diagnosis (−24 to −6 months, mean 7.9; 95% CI, 7.8–8.0: 6–0 months, mean 7.6; 95% CI, 7.4–7.8: 0–6 months, mean 7.7; 95% CI, 7.5–7.9: 6–12 months, mean, 7.8; 95% CI, 7.6–8.0: 12–24 months, mean 7.9; 95% CI, 7.7–8.1: 24–60 months, mean 7.8, 95% CI, 7.6–8.0). There were no changes in MEAN SYSTOLIC BLOOD PRESSURE (SBP; mm Hg) over 6 time periods from before to after cancer diagnosis (−24 to −6 months, mean 132; 95% CI, 131–133: 6–0 months, mean 132; 95% CI, 130–134: 0–6 months, mean 134; 95% CI, 132–136: 6–12 months, mean, 132; 95% CI, 130–134: 12–24 months, mean 131; 95% CI, 129–133: 24–60 months, mean 132, 95% CI, 130–134).	
Khan (2010) [17]		There was no difference between BC patients and controls in the PERCENT OF QUARTERS WITH BLOOD PRESSURE CONTROL (cancer, 62.8%; 95% CI, 58.9–66.6% versus 57.9%; 95% CI, 54.1–61.8%).	PC patients had a lower PERCENT OF QUARTERS WITH TOTAL CHOLESTEROL CONTROL than controls (cancer, 74.6%; 95% CI, 68.2–80.9% versus control, 83.7%; 95% CI, 78.6–88.7%).^a^
		There was no difference between colorectal cancer (CC) patients and controls in the PERCENT OF QUARTERS WITH BLOOD PRESSURE CONTROL (cancer, 63.7%; 95% CI, 57.7–69.7% versus control, 63.6%; 95% CI, 57.9–62.3%).	PC patients had a lower PERCENT OF QUARTERS WITH HBA1C CONTROL than controls (cancer, 63.7%; 95% CI, 57.2–70.4% versus control, 73.3%; 95% CI, 67.7–78.9%).^a^
		There was no difference between prostate cancer (PC) patients and controls in the PERCENT OF QUARTERS WITH BLOOD PRESSURE CONTROL (cancer, 67.9%; 95% CI, 61.7–74.0% versus control, 65.1%; 95% CI, 59.7–70.5%).	
		There was no difference between BC patients and controls in the PERCENT OF QUARTERS WITH TOTAL CHOLESTEROL CONTROL (cancer, 64.2%; 95% CI, 59.7–68.8% versus control, 70.4%; 95% CI, 66.1–74.6%).	
		There was no difference between CC patients and controls in the PERCENT OF QUARTERS WITH TOTAL CHOLESTEROL CONTROL (cancer, 75.3%; 95% CI, 69.1–81.6% versus control, 78.6%; 95% CI, 73.0–84.1%).	
		There was no difference between BC patients and controls in the PERCENT OF QUARTERS WITH HBA1C CONTROL (cancer, 69.6%; 95% CI, 59.6–68.7% versus control, 64.1%; 95% CI, 59.6–68.7%).	
		There was no difference between PC patients and controls in the PERCENT OF QUARTERS WITH HBA1C CONTROL (cancer, 72.5%; 95% CI, 66.0–79.1% versus control, 68.5%; 95% CI, 62.1–74.9%).	
Chiao (2010) [16]	MEAN HbA1c (%) was lower after compared to before CC diagnosis (mean before, 7.16; 95% CI, 6.9–7.4 versus mean after, 6.73; 95% CI, 6.5–7.0. Note that even though calculated confidence intervals overlap, authors report a *P*-value of 0.02-discrepancy probably because *P*-value based on paired *t*-test). MEAN LDL cholesterol (mmol/l) was lower after compared to before CC diagnosis (mean before, 95.6; 95% CI, 89.8–101.4 versus mean after, 85.7; 95% CI, 78.2–93.2. Note that even though calculated confidence intervals overlap, authors report a *P*-value of 0.04-discrepancy probably because *P*-value based on paired *t*-test).	MEAN DIASTOLIC BLOOD PRESSURE (mm Hg) was similar after compared to before CC diagnosis (mean before, 72.7; 95% CI, 70.8–74.6 versus mean after, 71.8; 95% CI, 69.8–73.8). MEAN SYSTOLIC BLOOD PRESSURE (mm Hg) was similar after compared to before CC diagnosis (mean before, 141.9; 95% CI, 137.4–146.4 versus mean after, 137.5; 95% CI, 134.2–140.8). TOTAL CHOLESTEROL (mmol/l) was similar after compared to before CC diagnosis (mean before, 165.6; 95% CI, 158.0–173.2 versus mean after 155.8; 95% CI, 146.3–165.3).	
Keating (2007) [19]	The PERCENT OF CANCER PATIENTS WHOSE MOST RECENT HBA1C WAS <8.0% was higher than controls (cancer, 73.4% versus control, 70.9% [insufficient data to calculate CIs]).	The PERCENT OF CANCER PATIENTS WHOSE MOST RECENT BLOOD PRESSURE WAS <130/80 mm Hg was similar to controls (cancer, 31.3% versus control, 32.2% [insufficient data to calculate CIs]).	The PERCENT OF CANCER PATIENTS WHOSE MOST RECENT LDL CHOLESTEROL WAS <100 mg/dl was lower than controls (cancer, 40.7% versus control, 42.2% [insufficient data to calculate CIs]).

^a^Authors reported these differences as statistically significant at *P* < 0.05. In the table, the calculated 95% confidence intervals for the proportions overlap. One of the corresponding risk ratios calculated and reported in Figure 2.5 was statistically significantly <1.0, and the other narrowly failed to meet the threshold for statistical significance. Therefore, it is likely that the authors assessed the statistical significance of differences between cancer patients and controls using the risk ratio approach.

Shaded results indicate that the findings were aggregated across multiple cancers, by the authors of that study, in instances where results from individual cancers included in that study also were reported.

#### Medication use and adherence

Seven studies reported on adherence to diabetes [24–27, 29, 30] and other [19] medications (Table 4). Measures of adherence, as well as the types of medications reported, differed substantially across the seven studies, and included proportions of patients receiving diabetes medications [29, 30], anti-hypertensives [19], and lipid-lowering agents [19], proportions of patients discontinuing current diabetes therapy [27], counts of diabetes medication discontinuation episodes [27], adherence to therapy [24, 26, 27] and persistence of therapy [25]. This diversity precluded presenting similar measures in plots, as was done for the other quality indicators described above.

**Table 4:** Medication use and adherence

	Difference between cancer patients and controls		
Study	Better in cancer than controls	No difference or inconclusive^a^	Worse in cancer than controls
	(or ‘after’ compared to ‘before’ cancer depending on the study design)	(or ‘after’ compared to ‘before’ cancer depending on the study design)	(or ‘after’ compared to ‘before’ cancer depending on the study design)
Yang (2016) [24]			Relative to the year before breast cancer (BC) diagnosis, the PERCENT OF PATIENTS ADHERING TO DIABETES MEDICATIONS (defined as a medication possession ratio [MPR] ≥80%) was lower during 1.5 years after diagnosis (before, 80%; 95% Confidence Interval [CI] 79–81% versus after, 53.1%; 95% CI, 51–55%).
Santorelli (2016) [25]		The ADJUSTED ODDS RATIOS (OR) FOR NON-ADHERENCE pre- post BC (or control date in controls) were not statistically significantly different between cancer patients and controls, when the proportion of days covered (PDC) was set at <70% (ratio of OR cancer to control = 1.24: *P* = 0.32), set at <80% (ratio of OR cancer to control = 1.35: *P* = 0.09), or set at <90% (ratio of OR cancer to control = 1.31: *P* = 0.07).	BC patients had increased ADJUSTED ODDS OF DIABETES MEDICATIONS ‘NON-ADHERENCE’ (PDC < 80%) compared to non-cancer controls (OR = 1.44: 95% CI, 1.07–1.95). The effect was similar when PDC threshold was changed to 70 and 90%. BC patients were more likely than controls to be ‘NON-PERSISTENT’ WITH DIABETES MEDICATIONS (adjusted Hazard Ratio [HR], 1.31: 95% CI, 1.04–1.66), where non-persistence was defined as discontinuation of diabetes medications.
Zanders (2015) [26]		BC patients experienced no change in MPR at the time of cancer diagnosis. However, they experienced a statistically significant monthly ongoing decline in MPR after the month of cancer diagnosis (−0.07% per month: 95% CI, −0.09 to −0.05%). Prostate cancer (PC) patients experienced a statistically significant increase in MPR at the time of cancer diagnosis (2.1%: 95% CI, 1.4–2.8%). However, they experienced a statistically significant monthly ongoing decline in MPR after the month of cancer diagnosis (−0.09% per month: 95% CI, −0.10 to −0.07%).	Overall, cancer patients (all types) experienced a statistically significant drop in MPR at the time of cancer diagnosis (−6.3%: 95% CI, −6.5 to −6.0%), and experienced a statistically significant monthly ongoing decline in MPR thereafter (−0.20% per month: 95% CI, −0.21 to −0.20%).
			Colorectal cancer (CC) patients experienced a statistically significant drop in MPR at the time of cancer diagnosis (−8.3%: 95% CI, −9.0 to −7.7%), and a statistically significant monthly ongoing decline in MPR thereafter (−0.17% per month: 95% CI, −0.19 to −0.16%).

			Oesophagael, stomach, pancreas or liver (OS) cancer patients experienced a statistically significant drop in MPR at the time of cancer diagnosis (−12.5%: 95% CI, −13.4 to −11.6%), and a statistically significant monthly ongoing decline in MPR thereafter (−0.45% per month: 95% CI, −0.47 to −0.42%).
			Pulmonary cancer (PuC) patients experienced a statistically significant drop in MPR at the time of cancer diagnosis (−15.2%: 95% CI, −16.0 to −14.4%), and a statistically significant monthly ongoing decline in MPR thereafter (−0.54% per month: 95% CI, −0.56 to −0.52%).
			Urinary cancer (UC) patients experienced a statistically significant drop in MPR at the time of cancer diagnosis (−0.8%: 95% CI, −1.5 to −0.1%), and a statistically significant monthly ongoing decline in MPR thereafter (−0.38% per month: 95% CI, −0.40 to −0.36%).
Calip (2015) [27]		Relative to the year before BC diagnosis (discontinuation episodes = 1.23; 95% CI, 1.11–1.35), the NUMBER OF DISCONTINUATION EPISODES was similar in each of four periods after cancer diagnosis: treatment period (1.06; 95% CI, 0.95–1.17); year +1 (1.16; 95% CI, 1.02–1.30); year + 2 (1.22; 95% CI, 1.07 to −1.37) and year +3 (1.97; 95% CI, 1.71–2.23). Relative to the year before BC diagnosis (proportion of patients discontinuing = 75%; 95% CI, 71%–78%), the PROPORTION OF PATIENTS DISCONTINUING DIABETES MEDICATIONS was similar in three of four periods after cancer diagnosis: treatment period (59%; 95% CI, 55–64%); year +1 (76%; 95% CI, 72–80%); year +2 (72%; 95% CI, 67–76%) and year +3 (71%; 95% CI, 66–75%).	Relative to the year before BC diagnosis (MPR = 86%; 95% CI, 84%–88%), diabetes MPR was lower in each of four periods after cancer diagnosis: treatment period (49%; 95% CI, 46–52%); year +1 (48%; 95% CI, 45–51%); year +2 (48%; 95% CI, 45–51%) and year +3 (52%; 95% CI, 49–55%). Relative to the year before BC diagnosis (proportion of adherent users [MPR ≥80%] = 75%; 95% CI, 72–79%), the PROPORTION OF DIABETES MEDICATION ADHERENT USERS was lower in each of four periods after cancer diagnosis: treatment period (25%; 95% CI, 21–28%); year +1 (27%; 95% CI, 23–31%); year +2 (24%; 95% CI, 20–28%) and year +3 (32%; 95% CI, 27–36%).
Shin (2014) [29]		There was no difference in the PERCENT OF PATIENTS RECEIVING DIABETES TREATMENT between cancer survivors (60.5%: 95% CI, 49.4–70.5%) and non-cancer, chronic disease controls (65.0%: 95% CI, 60.9–68.9%) or non-cancer, non-chronic disease controls (51.1%: 95% CI, 46.0–56.2%).	
Onitilo (2013) [30]			Patients with a history of BC were less likely to report METFORMIN USE than those without BC (cancer, 43%; 95% CI, 40–45% versus control, 58%; 95% CI, 55–61%).
			Patients with a history of PC were less likely to report METFORMIN USE than those without PC (cancer, 47%; 95% CI, 45–49% versus control, 58%; 95% CI, 55–61%).	
Keating (2007) [19]		There was no difference in the percent of cancer patients and controls RECEIVING ACE-I/ARB FOR HYPERTENSION (cancer, 76% versus control, 77% [insufficient data to calculate CIs]).	Cancer patients were less likely than controls to RECEIVE A STATIN FOR ELEVATED LOW DENSITY LIPOPROTEIN CHOLESTEROL (cancer, 77% versus control, 81% [insufficient data to calculate CIs]).

^a^Evidence from ‘before and after studies’ was considered inconclusive if differences in some of the intervals were statistically significant, while those in others were not.

Shaded results indicate that the findings were aggregated across multiple cancers, by the authors of that study, in instances where results from individual cancers included in that study also were reported.

Nonetheless, in contrast to other outcomes reported above, there was stronger evidence to indicate cancer had an adverse impact on adherence to medications, with 13/20 measures reported across the seven studies indicating adherence was statistically significantly lower in cancer patients than controls, and the majority of studies finding at least one instance in which medication adherence was either poorer after compared to before cancer diagnosis [24–27], or poorer in cancer patients compared to controls [19, 30]. Also, effect sizes were larger compared to other outcomes discussed above (Table 4).

## Discussion

Leading cancer organizations have expressed concern that overlooking other medical conditions during cancer treatment and follow-up could result in excess morbidity and mortality, thereby undermining gains associated with early detection and improved treatment of cancer [3, 4]. We conducted a systematic review to examine whether, among patients with diabetes, a diagnosis of cancer impacts the quality of diabetes care.

Overall, findings varied, both within and between studies, with most reporting a mixture of outcomes that fell into each of the three categories—no different, cancer better and cancer worse. Within the three quality indicator categories of healthcare visits, monitoring and testing of biologic parameters, and control of biologic parameters, no clear patterns emerged according to study design, patient population or methods of adjustment. Also, differences that were reported as statistically significant in the articles tended to be small and of questionable clinical relevance, as indicated by the narrow ranges of RRs (generally between 0.9 and 1.1) that were calculated. However, the results do indicate that cancer was associated with lower rates of medication use and adherence, with the majority (13/20) of measures in this quality indicator category showing cancer patients had lower rates than non-cancer controls.

### Strengths and limitations

This systematic review has several limitations. First, the searches were conducted only in Medline and Embase. Although findings from a previous study showed these two databases are sufficient for identifying English language papers on diabetes epidemiology [11], it is possible that additional articles/information would have been discovered had other databases such as the Cochrane Library [31], CINAHL [32] and PsycINFO [33] been included, if the search strategy had included grey literature resources, dissertations and theses, and conference proceedings [34], and if non-English language articles had been included. Second, the preliminary search produced in excess of 20 000 articles, and at that point the search was narrowed to those articles with both ‘cancer’ and any of ‘comorbid’ or ‘diabetes’ or ‘chronic’ in the title. An alternative approach would have been to review the titles, and possibly also the abstracts, of all 20 000+ articles in the preliminary search [35], which could have resulted in retaining articles that were inadvertently excluded when the search was narrowed based on the presence of key terms in the title.

Overall, the quality of the 15 studies, which were assessed using an established instrument [21], was high, and all but one study [14] met all of the applicable criteria for minimizing the risk of bias. High quality notwithstanding, another limitation is that there were insufficient data for performing a formal synthesis of outcomes across individual studies. First, even within the four broad categories of diabetes quality of care indicators defined for the systematic review, there was considerable heterogeneity in the study designs, patient populations, beginning of follow-up, duration of follow-up and definitions of the outcomes variables across the studies reporting those measures. Second, although visual inspection of the results showed little evidence of statistical heterogeneity, few studies reported sufficient data, e.g. sample sizes for the proportions reported, to perform a formal synthesis. For example, of the four studies [13, 14, 18, 20] that were used to calculate RRs of healthcare visits for cancer patients versus controls, only one [13] reported sufficient information on sample sizes. Of the six studies [15–17, 19, 27, 29] that reported on control of blood pressure [15–17, 19], cholesterol [15–17, 19], and/or HbA1c [15–17, 19, 27, 29], only one [17] provided sufficient data for a formal synthesis of the proportions of patients achieving control. Had a formal synthesis been feasible, it is possible more statistically significant differences in the quality of diabetes primary care indicators between the cancer cases and non-cancer controls would have been detected.

## Conclusion

There was no consistent evidence that, among patients diagnosed with diabetes, cancer adversely impacts healthcare visits, monitoring and testing of biologic parameters, or control of biological parameters. However, the evidence does indicate cancer is associated with poorer adherence to diabetes and other important medications.

Given several findings from a UK study of long-term survivors, and the fact that several recent studies have detected differences in outcomes, further primary research could be useful for examining the impact of incident cancer on a broad range of diabetes quality of care and outcomes indicators to address the concerns of cancer agencies in the UK.

## Supplementary Material

Supplementary Data
