# Supplementary material for: Quality of diabetes care in cancer: a systematic review
Source: Int J Qual Health Care. 2018 Jun 15;31(2):75–88. doi: 10.1093/intqhc/mzy124 (PMC6419905; doi:10.1093/intqhc/mzy124)
Supplement: Supplementary Data [file mzy124_griffiths_intqhc_supplemental_materials.docx]

**Supplemental Materials**

**Box A: Search Terms and Boolean Operators Used in Final Searches of Medline and Embase**

| **1** | exp Neoplasms/ |
| --- | --- |
| **2** | exp Carcinoma/ |
| **3** | exp Diabetes Mellitus, Type 1/ or exp Diabetes Complications/ or exp Diabetes Mellitus, Type 2/ or exp Diabetes Mellitus/ |
| **4** | exp Chronic Disease/ |
| **5** | exp Comorbidity/ |
| **6** | exp Quality Improvement/ or exp "Quality of Health Care"/ or exp Quality Assurance, Health Care/ or exp Quality-Adjusted Life Years/ or exp Quality Indicators, Health Care/ or exp "Quality of Life"/ or exp Total Quality Management/ |
| **7** | exp Disease Management/ |
| **8** | exp Disease Progression/ |
| **9** | 1 or 2 |
| **10** | 3 or 4 or 5 |
| **11** | 6 or 7 or 8 |
| **12** | 9 and 10 and 11 |
| **13** | Humans/ |
| **14** | exp Adult/ |
| **15** | 12 and 13 and 14 |
| **16** | Cancer.m_titl. |
| **17** | 15 and 16 |
| **18** | Diabetes.m_titl. |
| **19** | Comorbid.m_titl. |
| **20** | Chronic.m_titl. |
| **21** | Surviv.m_titl. |
| **22** | 18 or 19 or 20 or 21 |
| **23** | 17 and 22 |
| **24** | limit 23 to English language |

**Box B: Questions Used to Assess the Risk of Bias in Individual Studies**

| **Q1:** Were the inclusion/exclusion criteria similar across the comparison groups of the study? |
| --- |
| **Q2:** Were valid and reliable measures, implemented consistently across all study |
| participants, used to assess inclusion/exclusion criteria, intervention/exposure |
| outcomes, participant health benefits and harms, and confounding? |
| **Q3:** Were there reasonable attempts to balance the allocation between the groups or |
| match groups (e.g., through stratification, matching, propensity scores). |
| **Q4:** Were important confounding variables taken into account in the design and/or |
| other statistical adjustment such as instrumental variables)? |
| **Q5:** Are results believable taking study limitations into consideration? |

**Table A: Study Characteristics [continued on the following seven pages]**

|  | **Study Characteristics** | | | | | | |
| --- | --- | --- | --- | --- | --- | --- | --- |
| **Author Year Country** | **Study Design** | **Data Source** | **Patients** | **Enrolment Period** | **Length of Follow-up** | **Specific Outcomes Measures** | **Methods of Adjustment** |
| Yang  2016^24^  US | Cohort study of diabetes patients who were followed from before to after diagnosis of cancer. | National health care claims (insurance) database. | 3,424 women age >18 years, who were diagnosed with early-stage breast cancer (ESBC), and who obtained at least 1 diabetes prescription drug both before and after ESBC treatment. No control group. | First received a diagnosis of ESBC between January 1, 2009 and December 31, 2013. | From 1 year before ESBC diagnosis (index date) through 1.5 years after ESBC diagnosis, with a 6-month washout period after the index date. | Adherence to diabetes medications, defined as a medication possession ratio (MPR) ≥0.8, during the period after as compared to before the index date. MPR was defined as the ratio of the sum of the days' supply for all diabetes medication fills divided by the number of days between the first fill and the last fill plus the days' supply of the last fill. | Since adherence was compared before and after ESBC diagnosis, diabetes patients served as their own controls. |
| **Quality Indicators** |  |  |  |  |  |  |  |
| Medications |  |  |  |  |  |  |  |
| Santorelli 2016^25^  US | Cohort study of diabetes patients who were followed from before to after diagnosis of cancer (cases) or an assigned date for non-cancer controls. | Surveillance Epidemiology and End Results (SEER) multi-regional cancer registry data linked to Medicare health care claims. | 298 women, age ≥66 years, who were diagnosed with stage I-III breast cancer (BC), survived at least 2 years after BC diagnosis, had a diagnosis of diabetes during the year before BC diagnosis, and filled at least 2 prescriptions for an oral diabetes medication during the year before BC diagnosis. Control group of 1,192 women without cancer who met all the selection and cohort enrolment criteria and were frequency matched to BC patients by age and geographic area of residence at 4:1. | First received a diagnosis of BC (cases) in 2008, or were alive at the end of 2007 (controls). | From 1 year before BC diagnosis (cases) or assigned date (controls) through 2 years after the index date. | Non-adherence to oral diabetes medications, defined as having a proportion of days covered (PDC) <0.8. Sensitivity analysed at PDCs of 0.7 and 0.9. Medication non-persistence defined as being without diabetes medication for ≥26 days. | Comparisons of persistence and adherence between cancer patients and matched controls also adjusted for other factors. Adherence also was compared before and after BC diagnosis (cases) or assigned date (controls), so diabetes patients served as their own controls. |
| **Quality Indicators** |  |  |  |  |  |  |  |
| Medications |  |  |  |  |  |  |  |
| Zanders 2015^26^ Netherlands | Cohort study of diabetes patients who were followed from before to after diagnosis of cancer (cases) or an assigned date for non-cancer controls. | Eindhoven Cancer Registry (ECR) linked to the PHARMO (PHARmacoMOrbidity) Database Network. | 3,281 incident users of glucose-lowering drugs (GLDs), age >30 years, who were subsequently diagnosed with cancer (excluding non-melanoma skin). 12,891 matched, non-cancer controls. | First received GLDs between January 1, 1998 and December 31, 2011. | From start of GLDs to cancer diagnosis (cases) or assigned date (controls), and after the index date until the end of the data. Mean of 3.7 years before index date and of 6.6 years overall. | Adherence to diabetes medications, using MPR as the indicator. | Comparisons of adherence between cancer patients and matched controls. Adherence also compared before and after cancer diagnosis (cases) or assigned date (controls), so GLD patients served as their own controls. |
| **Quality Indicators** |  |  |  |  |  |  |  |
| Medications |  |  |  |  |  |  |  |
| Calip  2015^27^  US | Cohort study of diabetes patients who were followed from before to after diagnosis of BC. | Group Health Cooperative Commonly Used Medications and Breast Cancer Outcomes (COMBO) study. | 509 women who were diagnosed with early stage (I, II) invasive BC, and who had ≥1 dispensings of Group Health's first line diabetes medications in the year before cancer diagnosis. No control group. | First received a diagnosis of breast cancer between 1/1990 and 8/2007. | From 1 year before BC diagnosis up to the end of BC treatment plus 3 years. | Adherence to diabetes medications, defined as an MPR ≥0.8, during the period(s) after as compared to before the index date. Discontinuation rate, where discontinuation was defined as a ≥90-day gap between the end of the previous prescription's days' supply and the subsequent dispensing of the next diabetes medication. | Since adherence and persistence were compared before and after BC diagnosis, diabetes patients served as their own controls. |
| **Quality Indicators** |  |  |  |  |  |  |  |
| Medications |  |  |  |  |  |  |  |
| Heins  2015^28^  Netherlands | Cohort study of diabetes patients who were followed after diagnosis of cancer (cases) or after an assigned date for non-cancer controls. | NIVEL Primary Care Database. | 629 patients, age ≥55, who were diagnosed with non-skin cancer, had at least 2 years of follow-up after cancer diagnosis, and were diagnosed with diabetes before the end of 2 years after cancer diagnosis. Control group of 1,223 patients without cancer who met the other selection and cohort enrolment criteria, matched on age, sex, and practice. | First received a diagnosis of cancer between 1/2002 and 12/2010. | From 2 to 5 years after cancer diagnosis (cases) or assigned date (controls). | Annual general practitioner consultation rates: overall; diabetes; and other. | Comparisons between cancer patients and matched controls. |
| Shin  2014^29^  Korea | Cross-sectional cohort study of diabetes patients who were classified as cancer survivors, non-cancer chronic disease controls, or non-cancer non-chronic disease controls. | 2007-2011 Korea National Health and Nutrition Examination Survey (KNHANES). | 136 cancer survivors, 1,628 non-cancer chronic disease controls, and 896 non-cancer non-chronic disease controls, all age ≥30 years at diabetes diagnosis and time of survey. | Participated in KNHANES survey between 2007-2011. | Not applicable as this was a cross-sectional study. | Patient awareness of their diabetes status, defined as having been diagnosed with diabetes by a clinician. Diabetes treatment defined as being on pharmacological treatment for diabetes. Adequate glycaemic control defined as HbA1c <7%. | Multivariate comparisons of outcomes among the three groups, adjusting for age, sex, education, and body mass index. |
| Onitilo  2013^30^ Australia | Cross-sectional cohort study of diabetes patients who reported a history of BC or prostate cancer (PC), plus a control group consisting of diabetes patients without a history of cancer. | Patients identified within Australia's National Diabetes Services Scheme and surveyed for additional information, including history of cancer. | 158 patients, age ≥18, who reported a history of BC or PC. Control group consisted of 3,308 patients who did not report a history of cancer. | Completed a questionnaire in 2008. | Not applicable as this was a cross-sectional study. | Metformin use (yes/no). Self-reported HbA1c result. | Multivariate comparison of metformin use stratified by type of cancer, adjusted for age and duration of diabetes. |
| Snyder  2013^13^  US | Cohort study of cancer patients and controls with diabetes, who were followed after diagnosis of cancer (cases) or an assigned date for controls. | SEER national cancer registry data linked to Medicare health care claims. | 1,984 patients who were diagnosed with loco-regional BC, colorectal cancer (CC), or PC, were age ≥66 years at diagnosis, survived ≥3 years after diagnosis, and were identified as having diabetes within 1 year after diagnosis. 3,769 non-cancer controls who met the other eligibility criteria, were matched on age, race, and SEER region. | Diagnosed with cancer (cases) in 2004, or assigned an index date of January 1, 2004 (controls). | From day 366 through day 1,095 (3 years) after index date. | Visit every 6 months. Eye examination every year. HbA1c or fructosamine every 6 months. | Comparisons between cancer patients and matched controls. |
| Irizarry  2013^14^  US | Cohort study of patients with diabetes and cancer (cases), and a control group of diabetes and no cancer. | Health care claims (insurance) database. | Patients age ≥60 years diagnosed with diabetes and cancer (cases), or diabetes and no cancer (controls). Sample sizes not reported. | Not reported. | Outcomes assessed in 2005-2007. | Diabetes education. | None stated. |
| Bayliss  2011^15^  US | Cohort study of patients with diabetes followed from before to after diagnosis of BC, CC, or PC. | Diabetes registry within a single health maintenance organization. | 582 patients diagnosed with diabetes and subsequently diagnosed with BC, CC, or PC. No control group. | Diagnosed with diabetes between 1/1998 and 9/2008, and diagnosed with cancer ≥60 days later. | From prior to, up to 5 years after, cancer diagnosis. | Changes in HbA1c, systolic blood pressure (SBP), and LDL cholesterol over 6 periods from before to after cancer diagnosis. | Since HbA1c, SBP, and LDL cholesterol were compared before and after cancer diagnosis, diabetes patients served as their own controls. |
| Chiao  2010^16^  US | Cohort study of patients with diabetes followed from before to after diagnosis of CC. | Electronic medical records at a single medical center. | 122 patients diagnosed with diabetes and subsequently diagnosed with CC. No control group. | Diagnosed with cancer between 1/1999 and 12/2006. | From 1 year before, up to 1 year after, cancer diagnosis. | Changes in HbA1c, blood pressure, and cholesterol. Changes in primary care clinic and eye clinic visits. Changes in blood pressure, HbA1c, and cholesterol checks. All from 1 year before to 1 year after cancer diagnosis. | Since measures were compared before and after CC diagnosis, diabetes patients served as their own controls. |
| Khan  2010^17^  UK | Cohort study of patients with diabetes who were followed beginning ≥5 years after diagnosis of BC, CC, or PC, and a control group of patients with diabetes but no cancer. | General Practice Research Datalink. | 673 patients, age ≥30 years, with diabetes, who survived at least 5 years after diagnosis of BC, CC, or PC. 673 non-cancer controls meeting other eligibility criteria and matched to cancer patients on age, sex, and primary practice. | Entry in to the analysis from September 1, 2003 to August 30, 2006. | From the latter of 5 years after cancer diagnosis or September 1, 2003, to up to August 31, 2006. | Blood pressure, cholesterol, and HbA1c monitoring. Adequate control of blood pressure, cholesterol, and diabetes. | Comparisons between cancer patients and matched controls, with additional covariates included in multivariate analyses. |
| Hanchate 2010^18^  US | Cohort study of patients with diabetes who were followed after diagnosis of cancer, and a control group of patients with diabetes but no cancer | Pathology reports and tumor registry data linked to health care claims (insurance). | Subset of 422 patients, age ≥65 years, who were newly diagnosed with stage I-IIIA BC, who also had diabetes prior to cancer diagnosis. Control group comprised of a subset of 1,656 non-cancer controls meeting other eligibility criteria and matched to cancer patients on age, race, and location. Exact Ns for the diabetes subset not given. | 1997-1999. | Five years after cancer diagnosis or matched index date (controls). | Biennial lipid test, annual HbA1c test, biennial eye exam. | Comparisons between cancer patients and matched controls, with additional covariates included in multivariate analyses (latter not reported in paper). |
| Keating 2007^19^  US | Cross-sectional study of patients diagnosed with diabetes and cancer, and a control group of patients with diabetes but no cancer. | Clinical and administrative data from a large integrated health system. | 5,773 patients, age ≥21 years, who were diagnosed with diabetes by December 31, 2002, were alive through the end of 2003, and were diagnosed with invasive cancer during 1994 through 2001. Control group comprised of 23,092 non-cancer patients who met the other eligibility criteria. Propensity-matched cohort consisted of a subset of these. | 1994-2002 was the period used to establish both diabetes and cancer. | Outcomes assessed in 2003. | HbA1c test in past 6 months, most recent HbA1c <8.0%, LDL cholesterol test in past year, most recent LDL cholesterol <100 mg/dL, microalbumin test in past year, dilated retinal exam, most recent blood pressure <103/80 mm Hg for patients with hypertension, use of ACE I/ARB for patients with hypertension, use of statin for patients with elevated LDL cholesterol. | Comparisons between cancer patients and controls in propensity-matched subset. |
| Earle  2004^20^  US | Cohort study of patients diagnosed with diabetes and cancer, and a control group of patients with diabetes but no cancer. | SEER national cancer registry data linked to Medicare health care claims. | Subset of 14,884 patients, age ≥65, who were diagnosed with invasive CC in 1991 or 1992, who survived through the end of 1998, and who also had a diagnosis of diabetes before the beginning of the observation period (1997-1998). Control group comprised of a subset of 16,659 matched, non-cancer patients meeting the same eligibility criteria as the cancer patients. Control group matched on age, sex, race, and geographic location. | 1991-1996 (interpreted as end of qualification for diabetes subset). | Outcomes assessed in 1997-1998. | Visit every 6 months. Eye examination every year. HbA1c or fructosamine every 6 months. | Comparisons between cancer patients and matched controls. |

**Table B: Quality Scoring of Longitudinal Cohort Studies with a Control Group (Maximum Score of Eight Stars)**

|  | **Study** | | | | | | |  |
| --- | --- | --- | --- | --- | --- | --- | --- | --- |
|  | **Santorelli**  **2016^18^** | **Zanders**  **2015^19^** | **Heins**  **2015^21^** | **Snyder**  **2013^6^** | **Irizarry**  **2013^7^** | **Khan**  **2010^10^** | **Hanchate**  **2010^11^** | **Earle**  **2004^13^** |
| **Newcastle-Ottawa Criteria** |  |  |  |  |  |  |  |  |
|  |  |  |  |  |  |  |  |  |
| **Representativeness of the exposed cohort** |  | * | * |  |  |  |  |  |
| **Selection of the non-exposed cohort** | * | * | * | * | * | * | * | * |
| **Ascertainment of exposure** | * | * | * | * | * | * | * | * |
|  |  |  |  |  |  |  |  |  |
| **Comparability of cohorts** |  |  |  |  |  |  |  |  |
| Study controls for age | * | * | * | * |  | * | * | * |
| Study controls for additional factors | * | * | * | * |  | * | * | * |
| **Assessment of outcome** | * | * | * | * | * | * | * | * |
| **Follow-up long enough for outcomes to occur** | * | * | * | * | * | * | * | * |
| **Adequacy of follow-up of cohorts** | * | * | * | * | * | * | * | * |
|  |  |  |  |  |  |  |  |  |
| **Total Score** | **7/8** | **8/8** | **8/8** | **7/8** | **5/8** | **7/8** | **7/8** | **7/8** |

**Table C: Quality Scoring of Longitudinal Studies without a Control Group (Maximum Score of Five or Seven Stars†)**

|  | **Study** | | | | | | |
| --- | --- | --- | --- | --- | --- | --- | --- |
|  | **Yang**  **2016^17^** | **Calip**  **2015^20^** | **Shin**  **2014^22^** | **Onitilo**  **2013^23^** | **Bayliss**  **2010^8^** | **Chiao**  **2010^9^** | **Keating**  **2007^12^** |
| **Newcastle-Ottawa Criteria** |  |  |  |  |  |  |  |
|  |  |  |  |  |  |  |  |
| **Representativeness of the exposed cohort** |  |  |  |  |  |  |  |
| **Selection of the non-exposed cohort** | NA | NA | * | * | NA | NA | * |
| **Ascertainment of exposure** | * | * | * | * | * | * | * |
|  |  |  |  |  |  |  |  |
| **Comparability of cohorts** |  |  |  |  |  |  |  |
| Study controls for age | NA | NA | * | * | NA | NA | * |
| Study controls for additional factors | NA | NA |  | * | NA | NA | * |
| **Assessment of outcome** | * | * | * | * | * | * | * |
| **Follow-up long enough for outcomes to occur** | * | * | * | * | * | * | * |
| **Adequacy of follow-up of cohorts** | * | * | NA | NA | * | * | NA |
|  |  |  |  |  |  |  |  |
| **Total Score** | **4/5** | **4/5** | **5/7** | **6/7** | **4/5** | **4/5** | **6/7** |

†Either longitudinal cohort studies with a pre- post- design (maximum five stars) or cross-sectional (maximum seven stars) studies

NA – not applicable, study was “before and after” and did not include a control group.

**Table D: Risk of Bias Within Studies [continued on the following page]**

|  | **Study** | | | | | | |
| --- | --- | --- | --- | --- | --- | --- | --- |
| **Criteria** | **Yang**  **2016^17^** | **Santorelli**  **2016^18^** | **Zanders**  **2015^19^** | **Calip**  **2015^20^** | **Heins**  **2015^21^** | **Shin**  **2014^22^** | **Onitilo**  **2013^23^** |
|  |  |  |  |  |  |  |  |
| **Q1: Were the inclusion/exclusion criteria similar across the comparison groups of the study?** | NA | * | * | NA | * | * | * |
| **Q2: Were valid and reliable measures, implemented consistently across all study participants, used to assess inclusion/exclusion criteria, intervention/exposure outcomes, participant health benefits and harms, and confounding?** | * | * | * | * | * | * | * |
| **Q3: Were there reasonable attempts to balance the allocation between the groups or match groups (e.g., through stratification, matching, propensity scores)?** | NA | * | * | NA | * | * | * |
| **Q4: Were important confounding variables taken into account in the design and/or other statistical adjustment such as instrumental variables?** | NA | * | * | NA | * | * | * |
| **Q5: Are the results believable taking study limitations into consideration?** | ***** | ***** | ***** | ***** | ***** | ***** | ***** |

|  | **Study** | | | | | | | | |
| --- | --- | --- | --- | --- | --- | --- | --- | --- | --- |
| **Criterion** | **Snyder**  **2013^6^** | **Irizarry**  **2013^7^** | **Bayliss**  **2010^8^** | **Chiao**  **2010^9^** | **Khan**  **2010^10^** | **Hanchate**  **2010^11^** | **Keating**  **2007^12^** | **Earle**  **2004^13^** |  |
|  |  |  |  |  |  |  |  |  |  |
| **Q1: Were the inclusion/exclusion criteria similar across the comparison groups of the study?** | * | * | NA | NA | * | * | * | * |  |
| **Q2: Were valid and reliable measures, implemented consistently across all study participants, used to assess inclusion/exclusion criteria, intervention/exposure outcomes, participant health benefits and harms, and confounding?** | * | * | * | * | * | * | * | * |  |
| **Q3: Were there reasonable attempts to balance the allocation between the groups or match groups (e.g., through stratification, matching, propensity scores)?** | * |  | NA | NA | * | * | * | * |  |
| **Q4: Were important confounding variables taken into account in the design and/or other statistical adjustment such as instrumental variables)?** | * |  | NA | NA | * | * | * | * |  |
| **Q5: Are the results believable taking study limitations into consideration?** | ***** | ***** | ***** | ***** | ***** | ***** | ***** | ***** |  |

NA – not applicable, study was “before and after” and did not include a control group.

**Figure A: Risk Ratios for Health Care Visits**

*Snyder, 2013; **Irizarry, 2013; ***Hanchate, 2010; ****Keating, 2007; *****Earle, 2004. Triangles are point estimates. Bars are 95% Confidence Intervals.

**Figure B: Rates of Cancer Patients with a Health Care Visit or Monitoring, Before Versus After Cancer**

*Heins, 2015; **Chiao, 2010. Triangles are point estimates. Bars are 95% Confidence Intervals.

**Figure C: Calculated Risk Ratios for Monitoring and Testing**

*Snyder, 2013; **Khan, 2010; ***Hanchate, 2010; ****Keating, 2007; *****Earle, 2004. HbA1c, Glycosylated haemoglobin. LDL, Low density lipoprotein.

**Figure D: Calculated Risk Ratios for Control of Blood Pressure, Cholesterol, and HbA1c**

*Khan, 2010; **Keating, 2007. Triangles are point estimates. Bars are 95% Confidence Intervals.
